# Reaching the “Last Mile”: Fresh Approaches Needed for Guinea Worm Eradication

**DOI:** 10.4269/ajtmh.21-0433

**Published:** 2021-04-28

**Authors:** Claire J. Standley, Jordan Schermerhorn

**Affiliations:** Center for Global Health Science and Security, Georgetown University, Washington, District of Columbia

The global initiative to eradicate Guinea worm disease, which is caused by the parasite *Dracunculus medinensis*, has recently pushed back its target date from 2020 to 2030.^1^ This represents the fifth deadline extension since the effort began in earnest in the mid-1980s.^2^ The eradication program has long been a compelling story in public health because of its remarkable success considering the lack of medical countermeasures for the disease and also for its failure to finish the job. Emergent challenges have included newly discovered localized transmission in Angola, a major surveillance failure of 10 years without reported cases in Chad despite genomic evidence of ongoing circulation during that timeframe, and, most importantly, widespread uncontrolled transmission in animal populations, most notably among dogs in Chad.^3–5^

In *Surveillance of Human Guinea Worm in Chad, 2010–2018*, the authors provide an overview of Guinea worm patients in the country that currently has the highest known incidence in the world.^6^ The newly presented finding that active surveillance (where trained and supervised village-based volunteers routinely search for human and animal cases) was significantly associated with containment of human cases has major implications for program management and assumptions about the comprehensiveness of human case capture in official records. This builds on past evidence of the importance of robust case identification; in 2012, Cairncross et al. highlighted inadequate surveillance as a primary factor for stagnation in Ghana’s elimination program.^7^ The new work also describes barriers in border areas and among nomadic populations. Here, we detail some recommendations for new approaches intended to overcome challenges in the eradication effort.

First, the Carter Center has performed phenomenal work leading operational management of case detection and response in endemic countries, largely steering a reduction in Guinea worm disease incidence from an estimated millions to only tens of human cases per year. However, with new hurdles, the eradication campaign could benefit from fresh approaches, a critical eye, and diversity of thought. The polio eradication campaign, which has also encountered stumbling blocks, provides an example in the form of its Independent Monitoring Board (IMB).^8^ Established in part by the World Health Assembly, the polio IMB is provided full access to comprehensive surveillance data and is funded to conduct thorough site visits in endemic areas. Relatively little data regarding Guinea worm surveillance have been published, thus limiting external assessments of progress. Furthermore, a large portion of the eradication effort is privately funded, which may disincentivize accountability to partner governments and skew the objective assessment of progress. An independent monitoring board for Guinea worm eradication, removed from pressure to report success, could provide accountability.

Second, the effort could benefit from bottom-up, field-sourced strategies and more locally informed analyses. A decentralized approach that balances local knowledge and fresh outside perspectives has proven useful in previous eradication efforts. In *Death of a Disease*, D.A. Henderson’s account of the smallpox eradication campaign, it was noted that: “There was no possibility for a detailed plan that could be used everywhere. Creativity and flexibility in every program were not only welcome, they were to be encouraged and successful new concepts communicated to others.”^9^ Anthropological and ethnographic approaches may be particularly helpful in Chad, where human movement and behaviors vary temporally and geographically along the primary rivers in highly endemic areas and may be associated with animal transmission. Capturing this type of contextual information and suggestions for interventions from local-level program staff and the communities most impacted could allow the eradication program to adapt programing to local epidemiological and environmental quirks in a superior manner.

Third, efforts toward horizontalization of eradication programming could provide opportunities for national health systems to take a stronger role within a One Health approach. The World Health Organization’s Roadmap for neglected tropical diseases 2021–2030, launched in January, 2021, specifically mentions the “emerging recognition” that Guinea worm infections in animals could sustain transmission in humans as an example of a “last mile” challenge to eradication.^10^ The Roadmap also highlights, across all neglected tropical diseases, the importance of cross-cutting measures and targets, and it emphasizes One Health as a critical lens through which to view integration. The target for eradication of Guinea worm is the certification of all countries as “free of transmission” by 2030; this implies that even if infections are not reduced to zero in animals, efforts are in place to ensure no transmission to humans. The Roadmap goes further, noting that a critical action is to “develop a scientific and operational protocol for elimination of infections in animals.” Finally, the Roadmap specifically recommends increasing engagement in research and operational questions around controlling, and eventually eliminating, animal infections. For integration and horizontalization of Guinea worm in Chad in particular, considering the constraints to passive surveillance noted by Guagliardo et al.,^6^ one place to start may involve training rabies vaccination teams to identify symptoms and preserve worm samples from dogs outside of areas where proactive case searches are routinely conducted.^11^

In the context of a global pandemic, eradication efforts for a highly localized disease may seem like an abstract wish untethered to reality. They linger between the priorities of a global crisis and the often more pressing realities of inadequate health systems and more prevalent infectious disease threats.

Progress toward Guinea worm disease eradication in these contexts provides more broadly applicable benefits. It can reveal concrete evidence of elements of a public health system operating in tandem, demonstrating that cases can be found, treated, and reported up the chain, and that community health workers are sufficiently empowered to report honest accounts, including bad news, to their superiors. In summary, data from eradication efforts—especially those operating under extremely adverse circumstances—indicate functioning health systems able to capture the incidence of disease for endemic as well as emerging threats in addition to the eradication targets themselves. Reinforcing the Guinea worm disease eradication effort with external accountability, diversity of approaches, and sustainable horizontalization would honor the goal of providing equitable health systems to at-risk populations while simultaneously preparing those communities for future challenges.
